# Cleft Palate

**Published:** 1869-09

**Authors:** 


CLEFT PALATE.—Instrument for facilitating the Operation of Staphytoraphy.—The desirability of performing the operation for cleft palate at an earlier age than has hitherto been done can not be denied. The author has invented an instrument by which the patient is rendered perfectly helpless, the tongue being depressed and the mouth held wide open. By this means, and by the aid of chloroform, the defective formation may be remedied early in life, before the indistinct and nazal habit of speaking becomes permanently acquired. The same instrument will be found useful in excision of the tonsils in unruly children, and in operations on the pharynx and interior of the mouth. (Mr. T. Smith, p. 159.)

